# Investigating the Mechanistic Differences of Obesity-Inducing *Lactobacillus kefiranofaciens* M1 and Anti-obesity *Lactobacillus mali* APS1 by Microbolomics and Metabolomics

**DOI:** 10.3389/fmicb.2020.01454

**Published:** 2020-07-08

**Authors:** Yu-Chun Lin, Yung-Tsung Chen, Kuan-Yi Li, Ming-Ju Chen

**Affiliations:** ^1^Livestock Research Institute, Council of Agriculture, Executive Yuan, Tainan, Taiwan; ^2^Department of Animal Science and Technology, National Taiwan University, Taipei, Taiwan; ^3^Institute of Biotechnology, National Taiwan University, Taipei, Taiwan

**Keywords:** *Lactobacillus kefiranofaciens* M1, *Lactobacillus mali* APS1, obesity, microbolomics, metabolomics

## Abstract

Many studies have investigated the anti-obesity effects of probiotics in animal models and humans. However, few studies have focused on the mechanisms of obesity-inducing probiotics. In a previous study, we demonstrated that specific bacterial strains isolated from kefir, *Lactobacillus kefirnofaciens* M1 and *Lactobacillus mali* APS1, possess obesity and anti-obesity effects, respectively, in high-fat diet (HFD)-induced obese mice. Thus, in the present study, we systematically investigated whether APS1 and M1 affect energy homeostasis and lipid metabolism in HFD-induced obese mice and how this might be achieved. We observed that the M1/APS1 intervention influenced fat accumulation by regulating adipogenesis and inflammation-related marker expression both *in vitro* and in a HFD induced C57BL/6J mice model. We also observed putative links between key taxa and possible metabolic processes of the gut microbiota. Notably, families *Christensenellaceae* and *S24_7* were negatively correlated with body weight gain through increase in the essential esterized carnitine for energy expenditure. These results suggest the importance of specific probiotic interventions affecting leanness and obesity of subjects under a HFD, which are operated by modulating the tripartite relationship among the host, microbiota, and metabolites.

## Introduction

Diet-caused dysbiosis is an important contributor affecting the development of obesity by suppressing the metabolic capacity of gut microbiota and creating a chronic state of inflammation (Malaisé et al., 2017; Wilkins et al., 2019; Amabebe et al., 2020). Impaired gut permeability caused by dysbiosis leads to a continuous translocation of bacteria, which further jeopardizes the metabolism of nutrients and affects energy extraction, expansion and storage (Pindjakova et al., 2017; Nagpal et al., 2018; Amabebe et al., 2020; Tilg et al., 2020). Additionally, a low-grade activation of the innate immune system and a chronic state inflammatory response often accompany the excessive accumulation of lipid in overweight and obesity subjects due to physiological adaptive response to the stress of adipocyte (Shoelson et al., 2007; Reilly and Saltiel, 2017).

Clear evidence shows that the gut microbiome plays a crucial role in the functioning of the digestive tract and harvesting energy from the diet as well as modulating the immune system (Bäckhed et al., 2004; Maruvada et al., 2017; John et al., 2018; Zhang et al., 2019; Crovesy et al., 2020). Studies of gut microbiome in both animal models and humans revealed that bacterial species from the Bacteroidetes and Firmicutes phyla are dominated, which comprise more than 90% of the gut microbiota (Huttenhower et al., 2012; Hall et al., 2017). Besides, phyla of Actinobacteria, Proteobacteria and Verrucomicrobia are three important phyla based on the species level and their relative abundances (Mack et al., 2016; Hall et al., 2017).

Microbiome-targeted therapies such as probiotics, prebiotic-resistant starches, and fecal microbiota transplant (FMT) provide novel opportunities to prevent and treat the development of obesity and metabolic syndrome by manipulation of the gut microbiome (Kamada et al., 2013; Hill et al., 2014; Parekh et al., 2015; Maruvada et al., 2017). Accumulating evidence indicates that supplementation with specific probiotics in dietary interventions could affect the host metabolism and modulate the glucose homeostasis in an animal model and human studies (Holmes et al., 2012; Cano et al., 2013; Yadav et al., 2013; Cheng et al., 2015; Aoki et al., 2017). Some species within the *Lactobacillus* demonstrated anti-obesity (Karimi et al., 2015; Li et al., 2016) or anti-diabetic (Honda et al., 2012; Li et al., 2016) effects in animal models or humans.

Two strains, *Lactobacillus kefirnofaciens* M1 (M1) and *Lactobacillus mali* APS1 (APS1), were isolated from Taiwanese kefir grain and sugary kefir grain, respectively, in our lab (Chen et al., 2008). *L. kefirnofaciens* M1 has been demonstrated to have an immune-modulating activity *in vitro* (Hong et al., 2009) and anti-allergic (Hong et al., 2010), anti-asthma (Hong et al., 2011), and anti-colitis (Chen et al., 2012) effects in a murine model. The immunoregulatory effects of *L. kefirnofaciens* M1 involve upregulating the regulatory T (Treg) cell and inhibiting secretion of proinflammatory and inflammatory cytokines. *L. mali* APS1 demonstrated a beneficial effect on accelerating weight loss (Chen et al., 2018b) and on ameliorating hepatic steatosis (Chen et al., 2018a) in a murine model of diet-induced obesity. *L. mali* APS1 also possesses an anti-colitis effect. For intestinal barrier protection, both *L. kefirnofaciens* M1 and *L. mali* APS1 could improve epithelial barrier function *in vitro* by increasing the transepithelial electrical resistance (TEER) and significantly upregulating the level of the chemokine CCL-20 (Chen et al., 2012). The features of both strains suggest a potential to modulate chronic inflammatory activities and strengthen the gut epithelial layer of obese individuals. Surprisingly, M1 and APS1 exhibited opposite results on body weight and glucose homeostasis in high-fat diet (HFD)-induced obese mice. M1 possesses obesity effects, and APS1 has anti-obesity and anti-T2D effects (Lin et al., 2016b).

Many studies have investigated the anti-obesity or anti-diabetic effects of probiotics in animal models or humans. However, few studies have focused on the mechanisms of obesity-inducing probiotics. Thus, in the present study, we further systematically investigated whether APS1 and M1 affected energy and glucose homeostasis in HFD-induced obese mice by regulating adipogenesis and inflammation-related proteins through modulating metabolites *via* manipulating the gut microbiota composition.

## Materials and Methods

### M1 and APS1 Preparation

*L. kefiranofaciens* M1 and *L. mali* APS1 were grown in MRS broth (Difco Laboratories, Detroit, MI) and incubated at 37 and 30°C for 36 and 12 h, respectively. To prepare the probiotic treatments, the bacterial cells were harvested by centrifugation (3,000 × *g* for 15 min) during the log phase. Cultures were washed and suspended in saline three times. After washing, the bacteria were resuspended in saline and adjusted to 5 × 10^8^ CFU/ml for following *in vitro* and *in vivo* studies. The preparation of bacterial cell-free excretory supernatants (CFSs) and intracellular cell-free extracts (CFEs) was adopted from Zeng et al. (2016). Briefly, M1 and APS1 strains were harvested by centrifugation at 3,000 × *g* for 10 min, washed three times and resuspended in phosphate-buffered saline (PBS; adjusted to 10^9^ CFU/ml). The cell pellets of M1 and APS1 were resuspended before ultrasonic disruption (Mucroson XL 2000, Minsonix Inc., Farmingdale, NY, USA) in 3-s pulses for 30 s in an ice bath. The cell fractions were separated by ultracentrifugation at 3,000 × *g* for 10 min.

### *In vitro* Cell Culture

#### Cell Preparation

Both RAW264 macrophage cell line and 3T3-L1 preadipocytes, purchased from Bioresource Collection and Research Center, Food Industry Research and Development Institute (Hsinchu, Taiwan), were cultured in Dulbecco’s Modified Eagle’s Medium (DMEM; Corning, Lowell, MA, USA) containing 10% fetal bovine serum (FBS) and 1% antibiotic-antimycotic in a humidified atmosphere of carbon dioxide (CO_2_) 5/95% air atmosphere at 37°C.

#### 3T3-L1 Adipocyte Differentiation

3T3-L1 was first cultured in preadipocyte medium containing DMEM media with 10% bovine calf serum 25 mmol/L glucose, 10% bovine serum, 100 units/ml penicillin, and 100 μg/ml streptomycin in a humidified incubator set to 37°C and 5% CO_2_. Differentiation of 3T3-L1 preadipocytes into mature adipocytes was performed using a 3T3-L1 differentiation kit (Biovision, Milpitas, CA, USA) according to the manufacturer’s instructions. For initiate differentiation, cell cultures were replaced medium with differentiation medium containing differentiation cocktail and incubated for 3 days in a humidified incubator at 37°C with 5% CO_2_. After differentiation induction, cell cultures were maintained in a maintenance medium containing 1 μl of insulin to 1 ml of DMEM/F12 (1:1) with 10% FBS and the medium replaced in every 2–3 days. After 7–10 days, lipid droplet accumulation was observed by light microscopy BX-50 (OLYMPUS, Tokyo, Japan).

#### Co-culture of M1/APS1, Adipocytes, and Macrophages

After differentiation, 3T3-L1 cells were cocultured with RAW264 using the Transwell inserting with a 3 μm porous membrane (Corning, Lowell, MA, USA) to separate adipocytes (lower well) from macrophages (upper well). After 24-h incubation, 10^7^ CFU/ml of L. kefiranofaciens M1/L. *mali* APS1 were co-cultured with upper layer RAW264, at 37°C for 24 h. The cells in the lower well were lysed in Trizol for further study. For CFS/CFE, to examine the effect of M1 and APS1 on the differentiation of preadipocytes into mature adipocytes, 3T3-L1 preadipocytes were treated with 10% of CFE or CFS of M1 and APS1 in culture medium during the differentiation period.

#### Oil Red O Staining of Lipid Droplets in 3T3-L1

3T3-L1 cells were washed with DPBS and fixed with 4.0% formaldehyde (Sigma-Aldrich, St. Louis, MO, USA). After being fixed, the cells were stained with Oil red O solution (Sigma-Aldrich) containing 0.5% Oil red O in isopropanol for 30 min at room temperature (RT) and then washed three times with distilled water. The appearance of lipid accumulation was visualized by bright-field microscopy BX-50 (OLYMPUS, Tokyo, Japan). The area ratio (%) of Oil red O staining was determined by ImageJ 1.3 image analysis software (National Institutes of Health).

### *In vivo* Animal Model

A total of 40 male (7-week-old) C57BL/6J mice were purchased from BioLasco Corp., Ltd. (Ilan, Taiwan). The tested mice were housed in cages (one mouse per cage) under a controlled RT and 12 h light-dark cycle and provided food and filtered water *ad libitum*. All animal husbandry and experimental activities were conducted in accordance with national legal requirements. The studies were conducted in compliance with relevant guidelines, approved by the Institutional Animal Care and Use Committee of Livestock Research Institute, Council of Agriculture, Taiwan (Approval No: LRI IACUC 106-32). After 1 week of acclimatization, the mice were divided into four groups (*n* = 10 per group): a normal diet (ND) group that received the control diet (D12450B; Research Diets, Inc., New Brunswick, NJ, USA), a HFD group that received a HFD (D12492; Research Diets, Inc.) with oral administrated PBS, an M1 intervention group that was simultaneously fed a HFD and daily intragastric administration of 10^8^ CFU/mouse *L. kefiranofaciens* M1, and an APS1 intervention group that was simultaneously fed a HFD and *L. mali* APS1 10^8^ CFU/mouse intragastrically. All groups started from 8 weeks of age and continued until 16 weeks of age. The energy content of the HFD consisted of 60% fat, 20% carbohydrates, and 20% protein (5.24 kcal/g). The body weight and food intake of all mice were measured once a week. At the end of the experiment, all of the tested mice were fasted for 8 h, then anesthetized with isoflurane and sacrificed for serum and tissue collection. For RNA extraction, the adipose tissue was freshly harvested and then frozen stored in RNA*later* (Applied Biosystems, Foster City, CA, USA).

### Quantitative Real-Time Polymerase Chain Reaction Analysis

To analyze adipogenesis-related gene expression *in vitro* and *in vivo* models, the total RNA was isolated from the samples using the SPLIT RNA Extraction Kit (Lexogen, Vienna, Austria) based on the product manual. Extracted total RNA was further purified with Ambion™ RNase Inhibitor (Applied Biosystems) and TURBO DNA-free™ Kit (Applied Biosystems), and then saved for further cDNAs synthesis. cDNAs were synthesized from 1 mg total RNA using a High-Capacity cDNA Reverse Transcription Kit with RNase Inhibitor (Applied Biosystems). cDNAs were then analyzed by qPCR using a Power SYBR Green PCR Master mix (Applied Biosystems), which is performed using a StepOnePlus Real-Time PCR System (Applied Biosystems) for qPCR analyses. Specific primer sequences were adopted from Breasson et al. (2017). Relative quantification of gene expressions was performed using the comparative Ct method and normalized by an internal control, glyceraldehyde-3-phosphate dehydrogenase (GAPDH). Results were expressed as fold difference relative to a relevant control sample.

### Immunohistochemistry

After anesthetizing, epididymal white adipose tissue (eWAT) of the test animal was collected and fixed in 4% formaldehyde. Tissues were then paraffin-embedded and sectioned at 5 μm thickness. For Immunohistochemistry (IHC), sections were heated to 62°C to remove wax and then rehydrated prior to treatment with blocking serum (PBS or 0.5% Triton X-100 with 5% serum) for 1 h at RT. The appropriate volume of diluted primary anti-F4/80 (#70076, Cell Signaling Technology, Inc., Danvers, MA, USA) or anti-CD11c (#45581, Cell Signaling Technology) antibody was added and incubated overnight at 4°C. After washing, the sections were incubated at RT with HRP-conjugated anti-Rabbit-IgG secondary antibody (#31460, Thermo Fisher Scientific) for 1 h. Positive cells were visualized with diaminobenzidine (DAB; TA-060, Thermo Fisher Scientific) and counterstained with Mayer’s hematoxylin under a light microscope SG-EX30 microscope (SAGE vision, Taiwan). Each group had four sections and each section had three visual fields.

### Analysis of SCFA

The fecal samples were collected at the end of animal study and immediately stored at −80°C. Fecal short chain fatty acids (SCFAs) were analyzed using gas chromatography-flame ionization detector (GC-FID) with a modified method described by Mehrpouya-Bahrami et al. (2017). Briefly, 30 mg of each fecal sample was weighed and suspended in 300 μl saturated NaCl solution, and then homogenized for approximately 2 min. Second, subsequently, the samples were acidified with 20 μl of 10% sulfuric acid (Sigma-Aldrich, St. Louis, MO, USA). After acidification, 400 μl of diethyl ether was added to the samples for SCFAs extraction, and then centrifuged for 15 min at 14,000 × *g* under 4°C. The supernatant was quantified using a GC-FID to measure acetate, propionate, and butyrate.

### Western Blot Analysis

The fresh epididymal adipose of the tested mice was frozen immediately in liquid nitrogen after collection and used for preparation of the whole cell lysate. Two microgram total protein from adipose tissue, was separated by SDS-PAGE using 4–20% Tris-glycine gels (Invitrogen). After electrophoresis, proteins were transferred onto polyvinylidene difluoride (PVDF; Millipore IPVH 00010) membranes by iBlot 2 Dry Blotting System (Invitrogen) and blocked with 5% BSA (UniRegion Bio-Tech, Taipei. Taiwan) in Tris-buffered saline with 0.1% Tween 20 (TBST) at RT for 1 h. The member was then washed three times for 5 min each with 5 ml PBS with Tween-20 (PBST). After that, the membranes were subsequently incubated overnight at 4°C with primary antibodies (1:1,000 dilute with 1X PBST and 5% BSA) used for immunoblotting as follows: acetyl-CoA carboxylase (ACC; C83B10; anti-ACC, #3676, Cell Signaling Technology, Inc.), anti-fatty acid synthase (anti-FAS, #3180, Cell Signaling Technology, Inc.) and anti-β-actin antibody (BA3R, Thermo Fisher Scientific). Further, the membrane was washed three times for 5 min with 5 ml TBST, and then incubated with Goat Anti-Rabbit IgG Antibody, Peroxidase Conjugated (Millipore AP132P at 1:1,000) and Goat anti-mouse IgG, F(ab’)2-HRP (Santa Cruz, sc-3697 at 1:1,000) in 5 ml of blocking buffer with gentle agitation for 1 h at RT. After being washed a further three times with TBST, the anti-specific protein was visualized by the enhanced chemiluminescence detection system (Millipore). Quantification of the protein level in the luminescent bands was performed by ImageJ software (NIH).

### Analysis of Satiety Hormones

Total gastric inhibitory peptide (GIP), total ghrelin, Peptide YY (PYY), pancreatic polypeptide (PP), leptin, and resistin levels in mouse serum were measured using the Milliplex® MAP Kit and mouse metabolic hormone 96-well plate assay (Millipore Corporation) according to the manufacturer’s instructions.

### DNA Extraction and Sequencing of Gut Microbiota

The bacteria genomic DNA in the fecal samples from the ND, HFD, HFDM1, and HFDAPS1 groups was extracted by a QIAamp DNA Stool kit (QIAGEN, Hilden, Germany) according to the manufacturer’s instruction. The bacterial 16S ribosomal RNA variable region V3–V4 was amplified by PCR using a primer with a sample-specific barcode (V3F: 5′-CCTACGGGNGGCWGCAG-3′; V4R: 5′-GACTACHVGGGTATCTAATCC-3′) for microbiome analysis as described previously (Chang et al., 2015). PCR products were employed for microbiome analysis on an Illumina MiSeq sequencing platform according to the manufacturer’s instructions. The raw reads were denoised and filtered using QIIME (v1.7.0) to remove sequences that containing a short variable region (<90 bp) or undetermined bases (>2 bases) to get effective tags in V3–V4 variable region. All 16S rRNA gene sequencing data were submitted to the NCBI’s sequence read archive (SRA) database under the accession number PRJNA634807. Operational taxonomic unit (OTU) clustering and species annotation of barcoded PCR amplicons from the various fecal samples were performed from representative sequences using UPARSE software (Version 7.0.1001^1^) and the Greengenes database based on ribosomal database project classifier (Version 2.2), respectively. Chao 1 and Shannon index of alpha diversity and partial least squares-discriminant analysis (PLS-DA) plots were measured with QIIME (Version v1.7.0) using R software (Version 2.15.3). The abundance of specific bacteria at the genus‐ and species-level at *p* < 0.05 was determined using the non-parametric Kruskal-Wallis test. Taxonomic cladograms were derived from a Linear discriminant analysis Effect Size (LEfSe) analysis with linear discriminant analysis (LDA) scores greater than 3 and significance at *α* < 0.05, as determined by Kruskal-Wallis test. To clarify a co-occurrence network of the predominant microbiota, we performed a bivariate correlation analysis for the 15 most abundant families and obesity-related indicators using Spearman’s correlation coefficient and R and SAS software version 9.4 (SAS Institute Inc., Cary, North Carolina, USA) with a value of *p* as 0.05. The correlation heatmaps between gut microbiota and inflammation markers and serum metabolites were generated by R and SAS software version 9.4 (SAS), and then created using GraphPad Prism software (Prism 7, GraphPad Software Inc., CA, USA).

### Non-Targeted Serum Metabolomics Profile Investigation

Blood plasma samples were collected from the tested mice and stored at −80°C until analysis. The metabolomic profile analysis was conducted by the commission service of the Metabolomics Core Laboratory at the Center of Genomic Medicine, National Taiwan University (Taipei, Taiwan) using ultraperformance liquid chromatography (LC; Infinity 1290, Agilent Technologies, CA, USA) coupled with a quadrupole-time of flight (Q-TOF) mass spectrometer (6540, Agilent Technologies, Santa Clara, CA, US) with electrospray ionization. The Acquity HSS T3 column (2.1 × 100 mm, 1.8 μm pore size; Waters, Milford, MA, USA) was used and maintained at 40°C. Metabolites were identified by analysis of the spectra of mass spectrometry (MS) using XCMS2 (Benton et al., 2008), TIPick (Ho et al., 2013), and Batch Normalizer (Wang et al., 2013) methods.

### Statistical Analyses

The values are expressed as the mean ± standard deviation. In addition to the gut microbiota, all statistical analyses were performed by GraphPad PRISM 7 (GraphPad Software, Inc.). Significant differences among the experimental results expect for relative bacterial abundance were estimated by a one-way ANOVA with *post hoc* Tukey’s test. A probability level *p* < 0.05 was considered statistically significant. Differences in the abundance of specific bacteria were determined using the non-parametric Kruskal-Wallis test.

## Results

### APS1 and M1 Interventions Affect TG Lipid Accumulation Through Modulating Adipogenesis, Lipogenesis, and Inflammation in 3T3-L1 Cells

**Figure 1:**
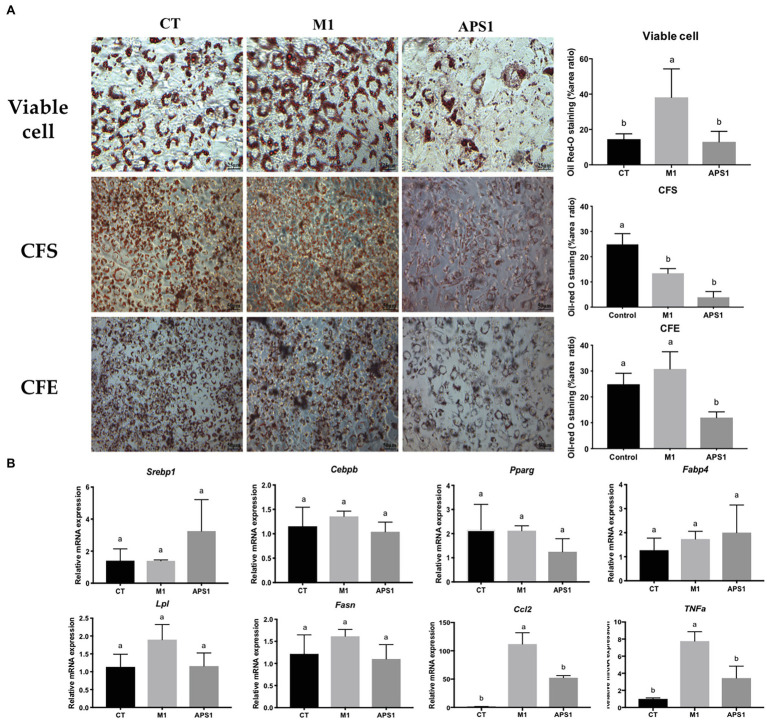
Effects of M1 and APS1 on modulation of adipogenesis in 3T3-L1 cells. **(A)** Lipid accumulation and the area ratio by ImageJ (%) in 3T3-L1 cells treated by viable cell, intracellular cell-free extract (CFE), bacterial cell-free excretory supernatant (CFS) of M1 and APS1 with Oil red O staining; and **(B)** modulation of adipogenesis and macrophage-derived *Tnf* and *Ccl2* relative mRNA levels in 3T3-L1 adipocytes after treated with viable M1 and APS1. Relative quantification of gene expressions was performed using the comparative Ct method with normalization to glyceraldehyde-3-phosphate dehydrogenase (GAPDH). Results were expressed as fold difference relative to a relevant control sample. Data are expressed as the mean ± SD (*n* = 3). Labeled means without a common superscript letter differ, *p* < 0.05.

After confirmation of no cytotoxicity effect on the cells (data no shown), we first *in vitro* evaluated the effect of M1 and APS1 on adipocyte differentiation and key lipogenesis markers involving in adipocytes. Results indicated that the M1 group in viable cells could induce significantly more production of lipid droplets than the CT and APS1 groups. Whereas, CFS and CFE from APS1 could significantly inhibit lipid accumulation relative to the control (*p* < 0.05; Figure 1A). Consistently, APS1 intervention showed a tendency to downregulate the mRNA expression of adipogenesis and lipogenesis markers (*Pparg*, *Lpl*, and *Fasn*) in 3T3-L1 cells when compared with the M1 group without significant difference (*p* > 0.05; Figure 1B). Conversely, treating cells with M1 promoted inflammation by upregulating biomarkers of inflammatory cytokines (*Ccl2* and *Tnf-α*; *p* < 0.05) (Figure 1B). *In vitro* results demonstrated that APS1 and M1 might affect lipid accumulation by modulating adipogenesis, lipogenesis, and inflammation.

### APS1 and M1 Interventions Affect the Obesity-Induced Mass/Size and Inflammation of Adipose Tissue by Regulating Adipogenesis, Macrophage Recruitment, and M1/M2 Status *in vivo*

**Figure 2:**
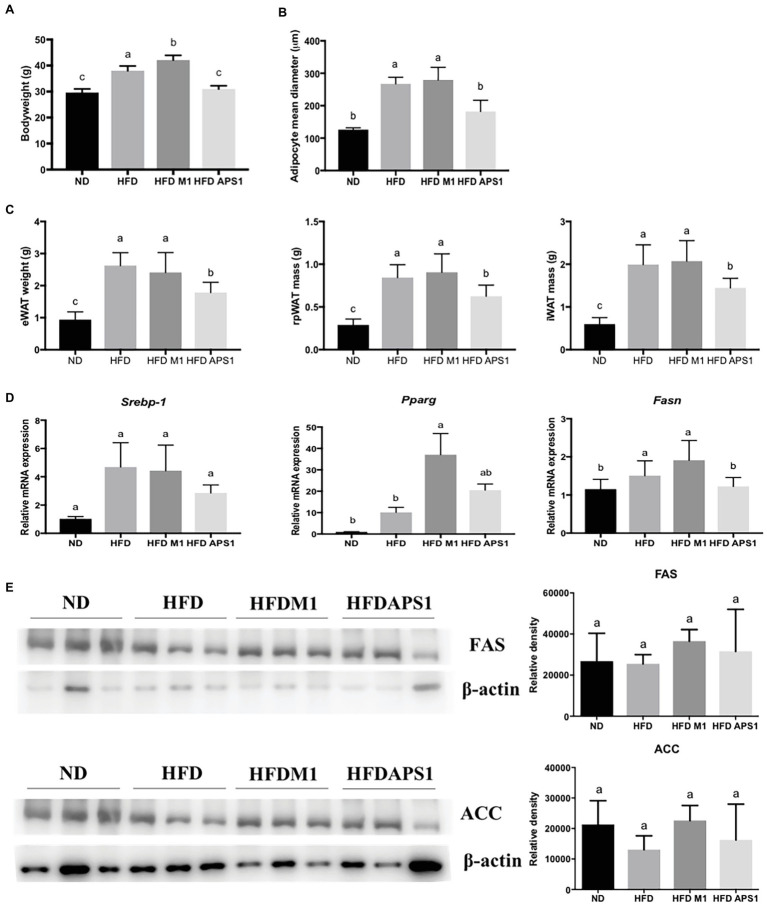
M1 and APS1 regulate fat hypertrophy and mRNA expressions of adipogenesis in mouse adipose tissue. M1 and APS1 interventions modulated **(A)** body weight; **(B)** the mean diameters of epididymal white adipose tissue (eWAT) adipocytes; **(C)** the weight of eWAT, rpWAT and iWAT; **(D)** adipogenesis-related mRNA expressions in mouse eWAT; **(E)** Western blot analyses of fatty acid synthase (FAS) and acetyl-CoA carboxylase (ACC) in adipose tissue. Relative quantification of gene expressions was performed using the comparative Ct method with normalization to GAPDH. Results were expressed as fold difference relative to a relevant control sample. Data are expressed as the mean ± SEM of 8–10 mice per group. Labeled means without a common superscript letter differ, *p* < 0.05.

*In vivo* results indicate that APS1 intervention (5 × 10^8^ CFU/mouse/day) not only significantly decreased the body weight and mean diameters of adipocytes, but also reduced the weights of eWAT (by 36.68%), rpWAT (by 28.53%), and iWAT (by 27.57%) relative to the HFD group (*p* < 0.05; Figures 2A–C). Whereas, the body weight of mice treated with M1 (10^8^ CFU/mouse) for 8 weeks was significantly higher than that of HFD mice (*p* < 0.05; Figure 2A). However, no significant difference was observed in mean diameters of adipocytes and the weights of retroperitoneal, intrascapular, epididymal, and visceral white adipose tissues between the HFDM1 and HFD groups (*p* > 0.05; Figures 2B,C).

We then investigated the possible underlying molecular mechanisms. Results showed that APS1 intervention could downregulate adipogenesis-related gene, *Fasn* (Figure 2D). Conversely, upregulation of adipogenic gene expression, *Pparg*, was observed in the HFDM1 group (Figure 2D). After studying mRNA expression, western blot analysis revealed that APS1 intervention showed a tendency to reduce the levels of FAS and ACC as compared with the HFDM1 group (*p* > 0.05; Figure 2E).

**Figure 3:**
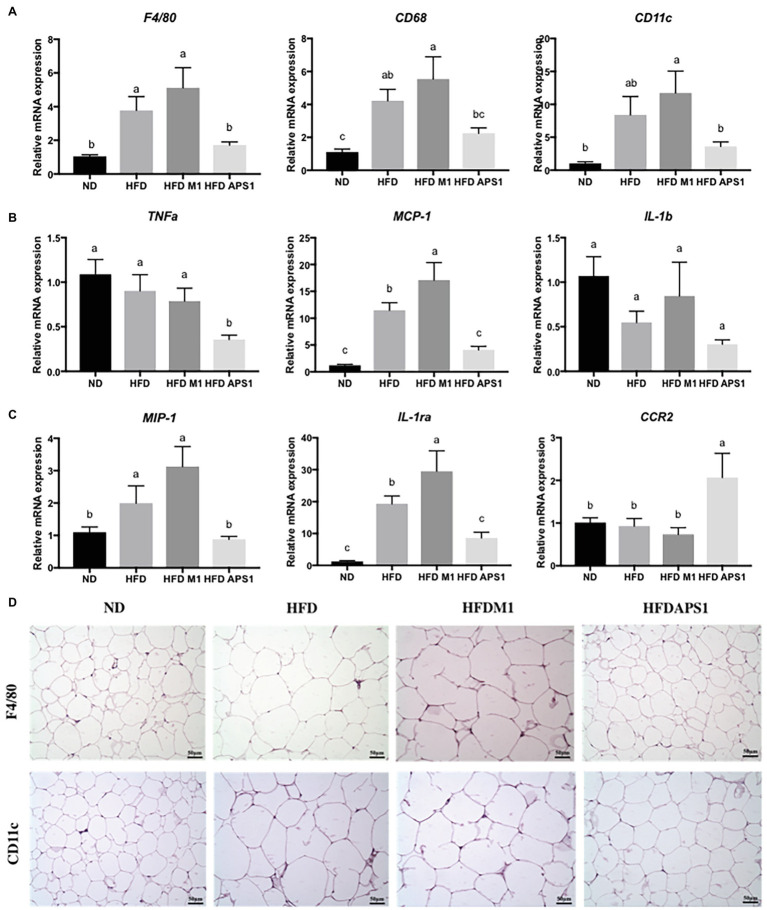
Effects of M1 and APS1 on expression of **(A)** macrophage markers; **(B)** inflammatory markers; **(C)** M1‐ and M2-activated macrophages and **(D)** immunohistochemistry (IHC) analysis of F4/80 and CD11c in adipose tissues of high-fat diet (HFD)-induced obese mice. Relative quantification of gene expressions was performed using the comparative Ct method with normalization to GAPDH. Results were expressed as fold difference relative to a relevant control sample. Data are expressed as the mean ± SEM of 8–10 mice per group. Labeled means without a common superscript letter differ, *p* < 0.05.

Besides adipogenesis, the effects of *L. kefiranofaciens* M1 and *L. mali* APS1 on types of macrophages accumulating on the adipose tissue of HFD-induced obese mice were also investigated. We found that APS1 intervention could significantly downregulate mRNA expressions of macrophage markers (F4/80, CD11c), inflammatory markers (*Tnf-α*, *MCP-1*), and M1 activated macrophage (*IL-1ra*), as well as upregulate M2 activated macrophage *CCR2* relative to the M1 and HFD groups (*p* < 0.05). Whereas, M1 intervention showed significantly higher mRNA expressions of macrophage markers (*CD68*, CD11c), inflammatory markers (*MCP-1*), and M1 activated macrophage (*IL-1ra*; *p* < 0.05) when compared with other groups (Figures 3A–C). The further IHC analysis for macrophage markers indicated that the fat subjects (HFD and HFDM1 groups) had larger adipocytes with higher F4/80 and CD11c in the eWAT than the lean subjects (NC and HFDAPS1 groups; Figure 3D).

### APS1 and M1 Interventions Regulate Metabolites and Fecal SCFAs Affecting Lipid Metabolism in HFD-Induced Obese Mice

**Figure 4:**
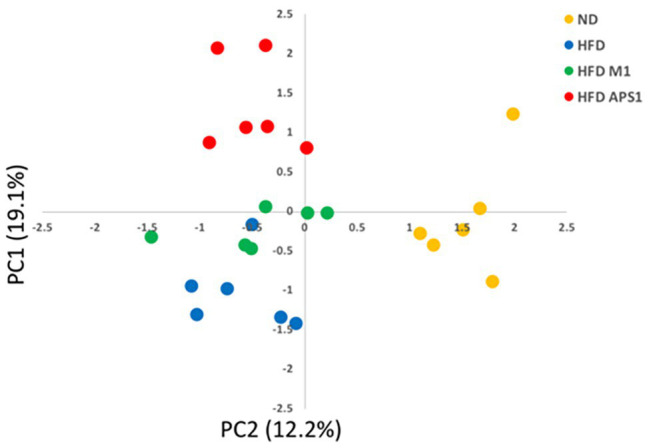
PCA plot of metabolomics profile based on PC1 and PC2 from mean intensity values of total detected metabolites.

Next, we studied the roles of metabolites and SCFAs specifically changed by the APS1 and M1 interventions on body weight gain and lipid metabolism. The PCA plot of non-targeted metabolomic profiling showed a significant separation among all tested groups using 24 significant detectable metabolites, explaining 31.3% of the variation (Figure 4). Among the metabolites, eight compounds, including 3-hydroxy 3-methylglutaric acid (HMG), corticosterone, glycerophosphocholine (GPC), glycine, l-isoleucine, octanoyl-l-carnitine (OLC), propionyl-l-carnitine (PLC), and traumatic acid, demonstrated significant differences among the four groups (*p* < 0.05; Table 1). After analysis by the Kyoto Encyclopedia of Genes and Genomes (KEGG) database, the metabolites were related to lipid metabolism, energy expenditure, lipid peroxidation, anti-inflammation, fat oxidation, and anti-oxidative stress (Table 1).

**Table 1:** The detected [by liquid chromatography–mass spectrometry (LC-MS)] differential metabolites of plasma sample among the normal diet (ND), high-fat diet (HFD), HFDM1, and HFDAPS1 groups.

Metabolites	Ion (m/z)	Mean intensity^1^,^2^				*p* value	Physiological function
		HFD/ND	ND/HFD	HFDM1/HFD	HFDAPS1/HFD		
3-Hydroxy 3-methylglutaric acid	161.046	0.097	10.271	3.286	3.912	0.00193	Lipid metabolism
Corticosterone	369.204	0.351	2.848	1.979	4.919	0.00634	Energy expenditure
Glycerophosphocholine	258.108	3.999	0.250	0.709	0.404	0.03595	Lipid peroxidation
Glycine	76.039	0.508	1.970	1.189	1.768	0.01156	Anti-inflammation
l-isoleucine	132.101	2.331	0.429	1.101	0.994	0.03404	Protein metabolism
Octanoyl-l-carnitine	288.217	0.078	12.755	1.186	1.977	0.00097	Fat oxidation
Propionyl-l-carnitine	218.139	0.502	1.992	1.189	1.243	0.00853	Fat oxidation
Traumatic acid	227.129	0.054	18.589	0.609	5.223	0.00108	Anti-oxidative stress

1 *n* = 6 per group.

2 Fold change was calculated by dividing the mean of the peak intensity of each metabolite from the ND, HFD, HFDM1, and HFDAPS1 groups.

**Figure 5:**
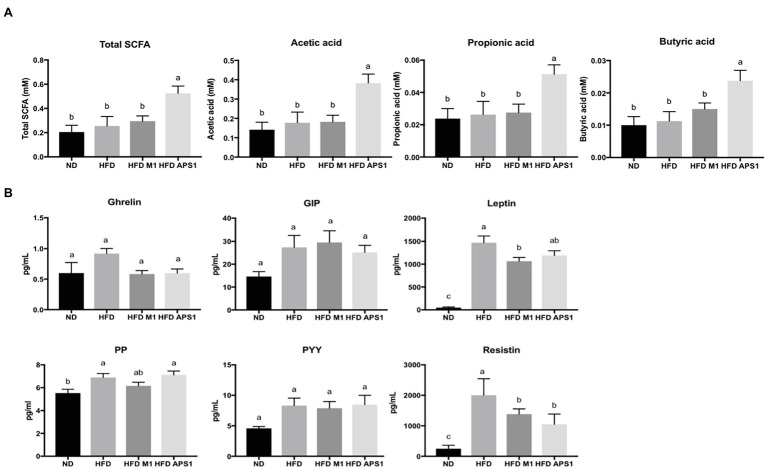
Effects of M1 and APS1 interventions on **(A)** fecal short chain fatty acids (SCFAs) excretion and **(B)** expression of satiety hormones in HFD-induced obese mice. (*n* = 8 per group). The data are expressed as the mean ± SEM. Labeled means without a common superscript letter differ, *p* < 0.05.

Besides, the mice fed with APS1 also had significantly increased excretion levels of cecal acetate, propionic acid and butyric acid (*p* < 0.05; Figure 5A). Upregulation of SCFAs was not observed in the HFDM1 group. Accompany the SCFAs, the HFD group showed significantly higher leptin, PP, and resistin than the ND group (*p* < 0.05; Figure 5B). The mice that received the APS1 significantly reduced the production of resistin compared to the HFD group (*p* < 0.05). Administration of M1 could downregulate the production of leptin and resistin (Figure 5B).

### APS1 and M1 Interventions Modulated Enriched Taxa in HFD-Induced Obese Mice

**Figure 6:**
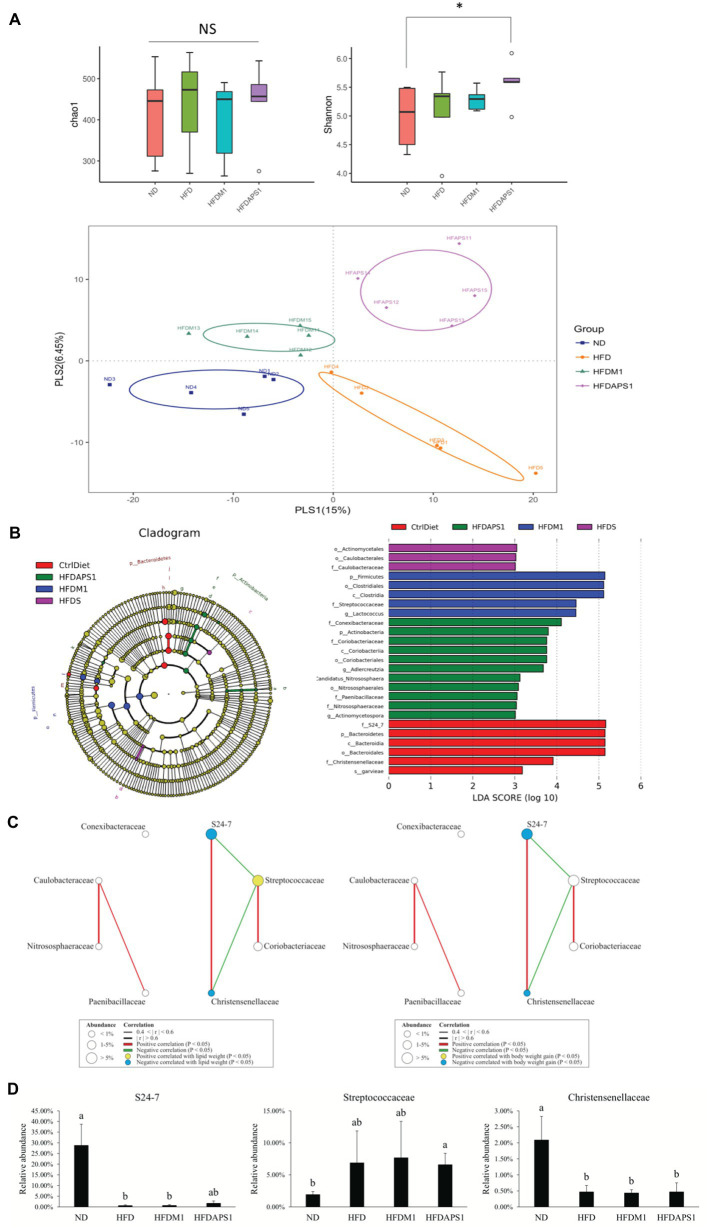
Effects of M1 and APS1 on manipulation of gut microbiota. **(A)** Alpha diversity of Chao1 richness index and Shannon’s diversity index and beta-diversity of supervised partial least squares-discriminant analysis (PLS-DA); **(B)** taxonomic cladograms derived from linear discriminant analysis effect size (LEfSe) analysis; bacterial networks co-occurring families of taxonomic cladograms correlating with **(C)** lipid weight and body weight gain; and **(D)** the relative abundance of FIGURE 6specific bacteria at the family level of taxonomic cladograms. LEfSe was used to compare the abundances of all detected bacterial taxa among four groups. The differentially abundant taxa shown in the histogram are significantly different by the Kruskal-Wallis test and have an linear discriminant analysis (LDA) score of greater than 3. *n* = 5 per group. Networks of co-occurring families showed associations among ND, HFD, HFDM1 and HFDAPS1. Circles symbols represent bacterial families positive (yellow) or negative (blue) correlated with body weight gain. The edges represent the strength of positive (green) or negative (red) correlations among the families. The data are expressed as the mean ± SEM. Labeled means without a common superscript letter differ. Values of *p* was calculated by KruskaleWallis test, ^✽^*p* < 0.05.

We then investigated whether the modulating effects of M1 and APS1 on features of obesity and adipose low-grade inflammation were associated with changes in metabolites *via* the gut microbiota using NGS. First, the alpha-diversity analysis of Chao 1 showed no significant difference (*p* > 0.05) in the richness of gut microbiota among the groups (Figure 6A). However, a significant Shannon diversity index (*p* < 0.05) was observed between the ND and HFDAPS1 groups (Figure 6A). Next, we performed a supervised PLS-DA based on OUT levels to evaluate the variant of gut microbial composition among the treated groups. Our PLS-DA plot shows that PLS1 and PLS2 explain 15 and 6.45% of the variation of gut microbiota composition, respectively, effectively separating each group (Figure 6A).

To further identify the specific bacterial taxa that were predominant as the biomarkers among the groups, the LEfSe was calculated. A total of 25 influential taxonomic clades (LDA score >3) were recognized (Figure 6B). The most impacted taxa in the ND group were the family *S24_7* (within the phylum Bacteroidetes), the order Bacteroidales (within the phylum Bacteroidetes), the family *Christenseneliaceae* (within the phylum Firmicutes), and species *Lactococcus garvieae* (within the phylum Firmicutes). After 8-week HFD feeding, the order Actinomycetales and family *Caulobacteraceae* (within the order Caulobacterales) were more predominant in the HFD group than in the other groups. Administrating APS1 specifically influenced 11 taxonomic clades in the mice, including the genera *Adlercreutzia* (within phylum Actinobacteria), *Actinomycetospora* (within phylum Actinobacteria), and the species *Candidatus nitrososphaera* (within phylum Actinobacteria). When orally administered M1, the most influential taxa were the class Clostridia (within the phylum Firmicutes), the family *Streptococcaceae* (within the phylum Firmicutes), and *Lactococcus* genus (within the phylum Firmicutes).

### M1 and APS1 Interventions Affect Body Weight Gain by Manipulating the Network of Co-occurring Predominant Bacteria at the Family Level

After identifying the specific bacterial taxa that were predominant as the biomarkers among four groups, the roles of these biomarkers in body weight gain and adipose inflammation were investigated. First, we constructed a module of the microbiome network among eight LEfSe selected families from all groups (Figure 6C). We observed that *S24_7* was positively correlated with *Christensenellaceae*. *Streptococcaceae* was negatively correlated with *S24_7* and *Christensenellaceae*. We further correlated eight LEfSe selected families with body weight gain and lipid weight. The families *S24_7* and *Christensenellaceae* showed a negative correlation with body weight gain and lipid weight while *Streptococcaceae* had a positive correlation with lipid weight. We validated these results by determining the relative abundance of specific bacteria at the family level of taxonomic cladograms, where we also found that the families *S24_7* and *Streptococcaceae* were enriched in lean and fat subjects, respectively (Figure 6D).

### M1 and APS1 Mediate Obesity-Related Adipose Inflammation by Regulating the Metabolites *via* Specific Bacterial Taxa

**Figure 7:**
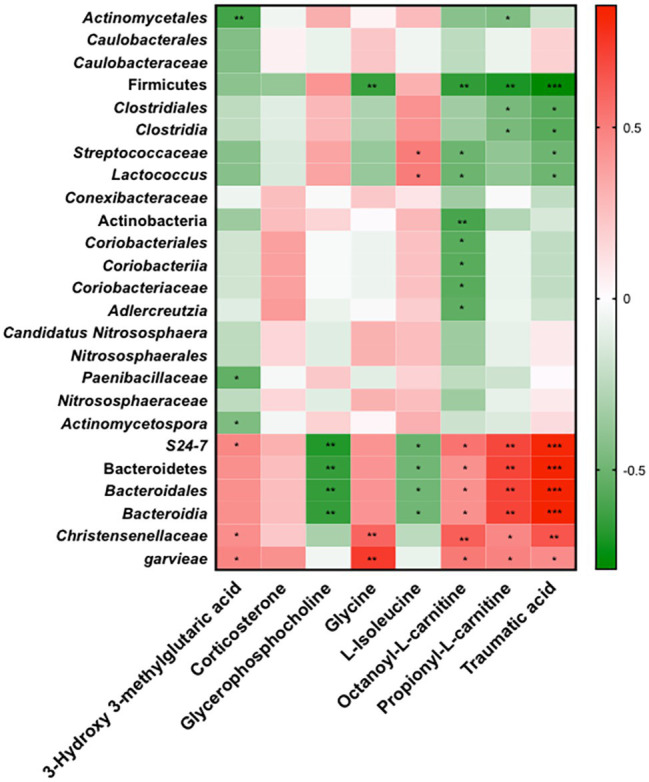
Spearman’s correlations of relative abundance of 25 LEfSe selected taxa with serum metabolites significantly modified in four groups. Red to green scale denote positive to negative associations. Spearman’s correlations were employed in agreement with data distribution and verified by Kruskal-Wallis test. ^*^p < 0.05, ^**^p < 0.01, ^***^p < 0.0001 following the Spearman’s correlations.

Finally, using Spearman’s correlations, we verified that M1 and APS1 modulate inflammation and lipid metabolism by regulating the metabolites *via* microbiota (Figure 7). Serum OLC, PLC, and traumatic acid showed positive correlations with families *S24_7* and *Christensenellaceae*, and species *garvieae*, and had negative correlations with phylum Firmicute, family *Streptococcaceae*, and genus *Lactococcus* (Figure 7). Circulating isoleucine was positively correlated with the *Streptococcaceae* family and *Lactococcus* genus and negatively correlated with families *S24_7* and *Christensenellaceae* in the whole study population (Figure 7). Four taxa, including *S24_7* and phylum Bacteroides, had significant negative correlations with serum GPC. Serum HMG showed negative correlations with family *Paenibacillaceae* and genus *Actinomycetospora*, but positive correlations with *S24_7*, *Christensenellaceae*, and *garvieae*.

## Discussion

In a previous study, we demonstrated that specific bacterial strains isolated from kefir, *L. kefirnofaciens* M1 and *L. mali* APS1, possess obesity and anti-obesity effects, respectively, in HFD-induced obese mice. In the present study, we investigated the mechanism contributing to the opposite effects in body weight, further underscoring the importance of the tripartite relationship among the host, microbiota, and metabolites. Our findings highlight three interdependent effects of the gut microbiome on adipogenesis, lipogenesis, and inflammation through APS1 and M1 interventions.

First, we observed that APS1 and M1 interventions elucidated different regulations on gene expression of lipid metabolism and cellular crosstalk in both cell line and adipose tissue. The HFDM1 group might be more effective at extracting energy from food and stimulating lipogenesis through upregulating an essential nuclear hormone receptor (*Pparg*). Conversely, the HFDAPS1 group suppressed lipid accumulation by downregulating the mRNA expression of adipose *Pparg* and *Fasn*, as well as observed tendency for reduced SREBP 1 and ACC at protein level. Adipocyte differentiation is regulated by complex modulation of various transcription factors and specific proteins. The PPARγ is considered key early regulators of adipogenesis, while FAS and ACC are the two major enzymes of *de novo* lipogenesis, which are abundantly expressed in adipose tissues under the control of SREBP1 (Nishimura et al., 2014; Moseti et al., 2016; Gross et al., 2017). SREBP1, the primary substrate for AKT-mediated lipid metabolism, promotes lipid biosynthesis and inhibits lipolysis (Niso-Santano et al., 2015; Yan and Zheng, 2017; Choi et al., 2018).

Besides, because of obesity, the HFDM1 group demonstrated low-grade inflammation in visceral adipose tissue through enhancing production of inflammation-related cytokines, *Ccl2* and *Tnf-α*. Additionally, low-grade inflammation also affects adipocyte function with macrophage infiltration of adipose tissue. The adipose tissue macrophages (ATMs) are characterized as proinflammatory macrophages (M1) and noninflammatory macrophages (M2) according to their polarization state (Lumeng et al., 2007; Chang et al., 2015). Obesity induces the accumulation of M1 ATMs, whereas, M2 ATMs predominate in lean mice (Kitade et al., 2012). In the present study, administration of *L. kefiranofaciens* M1 with HFD upregulated M1 activating marker (*IL-1ra*), leading to a proinflammatory environment (*MCP-1*) relative to the HFD group. Conversely, APS1 invention upregulated M2 activating macrophages in HFD-induced obese mice *via* significantly downregulating relative mRNA expressions of M1 macrophage and M1 activating markers (*MIP-1*, *IL-1ra*). Thus, our findings provide a possible link between probiotic intervention, obesity, and inflammation.

Secondary, we observed that the abundance of obesity‐ or inflammation-associated bacteria in intestinal microbiota was regulated by the APS1 and M1 interventions. Microbial diversity has previously been identified as a significant factor influenced by diet (Clarke et al., 2014), which is decreased among the overweight (Le Chatelier et al., 2013) and increased in lean individuals (Wang et al., 2010; Lu et al., 2016). The results of the Shannon diversity index and PLS-DA were consistent with previous findings. We further identified the key microorganisms associated with M1 and APS1 intervention, and ultimately, leading to the obese and lean animals. After analysis by LEfSe and Spearman’s correlations, two families, *S24_7*, and *Christensenellaceae*, biomarkers in the ND group, were negatively correlated with body weight gain and lipid weight. Both families have been reported to be associated with lean animals (Zhang et al., 2009; Serino et al., 2012; Lin et al., 2016a; Chen et al., 2018b; Peters et al., 2018), which were reduced in the HFDM1 and enriched in the HFDAPS1. The family *Streptococcaceae*, which was in abundance in obese animals (Peters et al., 2018), was abundant in the HFDM1 and reduced in the HFDAPS1. The recently studied also observed that *Christensenella minuta* and its close relatives in the *Christensenellaceae* family were the most heritable bacteria. People with higher amounts of the gut bacteria *C. minuta* tended to be leaner (Goodrich et al., 2014). Additionally, some members of the *S24_7* family could be differentiated by their degree of IgA labeling, indicating that *S24_7* might be targeted by the innate immune system (Ormerod et al., 2016).

Alternation of metabolites profile has been regarded as an indicator to physiological conditions, reflecting the changes of gut microbiota composition (Chen et al., 2019). In the present study, eight key metabolites in serum associated with M1 and APS1 interventions were identified by the non-targeted metabolomic profiling. HMG could effectively lower serum cholesterol (Lupien et al., 1979) and prevent hypertriglyceridemia in rats (Yousufzai and Siddiqi, 1977). PLC and OLC are essential substrates for energy expenditure. PLC is an SCFA esterified to carnitine that plays an important role in fatty acid oxidation and energy expenditure (Mingorance et al., 2012). OLC, which promotes free fatty acids (FFAs) or acyl-CoA being able to move across the inner mitochondrial membrane, has a vital role in fatty acid metabolism (Reuter and Evans, 2012). Isoleucine, a branched-chain amino acid (BCAA), suppressed the development of NAFLD among obese youth by reducing the ability of insulin on hepatic glucose production in adiposity (Perng et al., 2014; Butte et al., 2015; Tricò et al., 2017). GPCs could increase growth hormone secretion and hepatic fat oxidation, resulting in increased serum FFA levels in young adults (Kawamura et al., 2012). GPCs are also considered sensitive indicators for obesity-related diseases such as cardiovascular disease (CVD) in adults (Syme et al., 2016). After further analysis by Spearman’s correlations, we demonstrated that the family *Streptococcaceae* with an obese animal was positively correlated with l-isoleucine, and negatively corrected with OLC and traumatic acid, providing a possible link between *Streptococcaceae* and the development of obesity. Conversely, the families *S24_7* and *Christensenellaceae* were negatively correlated with GPC and l-isoleucine and positively corrected with HMG, glycine, OLC, PLC, and traumatic acid, contributing to the connection between these two families and leanness.

Besides the key serum metabolites, SCFAs also showed distinct actions relevant to various gut incretins, hormones, and energy homeostasis (Rosenbaum et al., 2015). Oral administration of butyrate significantly increases plasma levels of the GIP, glucagon-like peptide-1 (GLP-1), PYY, insulin, and amylin, which would have net effects of slowing digestion and nutrient intestinal transit, promoting satiety, and increasing plasma insulin. Acetate is reported to increase leptin release by fat cells. Butyric acid and propionate increase G-protein-mediated secretion of PYY and GLP-1 in the gut, and rates of lipolysis and lipogenesis in fat cells (Fujishima et al., 2012; Kimura et al., 2014). In the present study, oral administration of APS1 promoted the SCFAs production and downregulated the expressions of resistin and leptin in the intestine. This finding suggests that SCFAs were modulated by APS1-manipulated gut microbiota and further modulated the production of SCFAs to regulate the expression of the gut hormone, thereby controlling host appetite through the gut-brain axis. For M1 intervention, downregulating the expression of resistin, PP, and leptin in the intestine was also observed, indicating that the obese inducing effect of *L. kefiranofaciens* M1 is not due to increasing appetite. This result is paralleled with our previous finding in daily food intake.

Finally, by reviewing papers and our previous data, M1 and APS1 could exert effects on the gut microbiota through the following mechanisms. (1) M1 and APS1 could directly affect gut microbial colonization through inhibitory/promoting effects. Both strains could produce lactic acid and certain SCFAs, which were identified earlier as a key factor affecting microbiota. SCFAs could also interact with the host, contributing to reducing oxygen concentrations and creating a less favorable environment for pathogen growth (Rivera-Chávez et al., 2016). Effect of other metabolic byproducts produced by both strains on the gut microbiota still needs to be further investigated. (2) M1 and APS1 could also impact the gut microbiota indirectly by manipulating the immune system, which in turn influences the colonizing microbiota. Both strains have been reported to interact with immune cells through toll-like receptor (TLR) and modify the T helper (Th1,Th2) or T regulatory cytokines (Hong et al., 2010; Lin et al., 2016b), which plays crucial roles in host defense against colonization of certain microorganisms (Pickard et al., 2017). (3) Additionally, in our early study, *L. kefiranofaciens* could form biofilm (Wang et al., 2012), which might be related to quorum sensing system to increase the cell density against harsh environment. Quorum sensing is a communication mechanism between bacteria to control specific processes, such as biofilm formation, production of secondary metabolites and stress adaptation mechanisms. Gram-positive bacteria have been reported to synthesize small autoinducing peptides (AIPs) as distinct signaling molecules to mediate quorum sensing (Spangler et al., 2019).

## Conclusions

In the present study, we show that interventions involving *L. kefiranofaciens* M1 and *L. mali* APS1 with HFD affect adipogenesis, lipogenesis, and inflammation by regulating the expression of metabolites, and that this is achieved by manipulating microbiota. Administration of *L. kefiranofaciens* M1 and *L. mali* APS1 with HFD had different influences on the abundances of specific families, *Streptococcaceae*, *S24_7*, and *Christensenellaceae* in gut microbiota, which further diversifies the specific serum metabolites, GPC, l-isoleucine, HMG, glycine, OLC, PLC, and traumatic acid. The different levels of these metabolites could contribute to lean and obese subjects by regulating the metabolic pathways involved in adipogenesis, lipogenesis, lipid metabolism, and energy expenditure. SCFAs were also modified by APS1-manipulated gut microbiota to regulate the expression of gut hormones, thereby controlling host appetite through the gut-brain axis. Additionally, the M1 and APS1 interventions regulated low-grade inflammation and macrophages in adipose tissue, leading to obese-related proinflammatory macrophages and leanness-related noninflammatory macrophages, respectively. This study highlights the importance of specific probiotic interventions affecting leanness and obesity of the subjects under HFD, achieved by modulating the tripartite relationship among the host, microbiota, and metabolites.

## Data Availability Statement

The original contributions presented in the study are publicly available. This data can be found at the NCBI under accession number PRJNA634807.

## Ethics Statement

The animal study was reviewed and approved by Institutional Animal Care and Use Committee of Livestock Research Institute, Council of Agriculture, Taiwan (Approval No: LRI IACUC 106-32).

## Author Contributions

Y-CL drafted and prepared the initial article. Y-CL and Y-TC conducted a research and investigation process, performing the experiments and data collection. K-YL conducted the formal analysis on statistical techniques to analyze study data. M-JC were involved in the management and coordination responsibility for the research activity planning and execution. Y-CL prepared the published work by those from the original research group, specifically critical review and revision of pre-publication stages. All authors contributed to the article and approved the submitted version.

### Conflict of Interest

The authors declare that the research was conducted in the absence of any commercial or financial relationships that could be construed as a potential conflict of interest.

## Footnotes

^1^http://drive5.com/uparse/
